# Editorial for the Special Issue “Physical Medicine and Rehabilitation: Trends and Applications”

**DOI:** 10.3390/life15030419

**Published:** 2025-03-07

**Authors:** Karen Sui Geok Chua, Krisna Piravej, Jiunn-Horng Kang, Li-Wei Chou

**Affiliations:** 1 Institute of Rehabilitation Excellence (IREx), Tan Tock Seng Hospital Rehabilitation Centre, TTSH-Integrated Care Hub (ICH), 1 Tan Tock Seng Link, Singapore 307382, Singapore; karen_chua@ttsh.com.sg; 2 Department of Rehabilitation Medicine, Faculty of Medicine, Chulalongkorn University, Bangkok 10330, Thailand; kppmrchula@gmail.com; 3 Thai Red Cross Rehabilitation Center, Thai Red Cross Society, Samut Prakan 10280, Thailand; 4 Department of Physical Medicine and Rehabilitation, Taipei Medical University Hospital, Taipei 110301, Taiwan; jhk@tmu.edu.tw; 5 Graduate Institute of Nanomedicine and Medical Engineering, College of Biomedical Engineering, Taipei Medical University, Taipei 110301, Taiwan; 6 Department of Physical Medicine and Rehabilitation, China Medical University Hospital, Taichung 404332, Taiwan; 7 Department of Physical Therapy, Graduate Institute of Rehabilitation Science, China Medical University, Taichung 406040, Taiwan; 8 Department of Physical Medicine and Rehabilitation, Asia University Hospital, Asia University, Taichung 413505, Taiwan

## 1. Introduction

Physical medicine and rehabilitation blend scientific expertise with compassionate care to enhance quality of life and restore functionality for individuals with complex conditions. As the field rapidly grows due to an aging population, rising chronic disease rates, and technological advancements, its transformative impact continues to expand. This Special Issue of *Life*, entitled “Physical Medicine and Rehabilitation: Trends and Applications”, features 17 innovative papers that delve into the latest research, technologies, and therapeutic methods. Covering areas from stroke recovery and neuro-oncological rehabilitation to cutting-edge technologies and specialized interventions, these studies showcase the field’s diversity and depth, bridging clinical insights with advanced methodologies. Recent advancements in rehabilitation science have highlighted the increasing role of personalized treatment approaches, robotics, and digital health in rehabilitation outcomes [1]. Additionally, global estimates indicate a growing demand for rehabilitation services, with an urgent need to address disparities in accessibility and outcomes [2].

## 2. Themes and Highlights

### 2.1. Neurological Rehabilitation

Neurological rehabilitation is crucial for patients recovering from acquired brain injuries, including stroke, traumatic brain injury (TBI), brain tumors, and spinal cord injuries. The featured studies emphasize early intervention, personalized care, and caregiver involvement.

- Sheng Li et al. [3] integrated the International Classification of Functioning, Disability, and Health (ICF) framework into stroke rehabilitation treatments. Their systematic review demonstrated that the ICF framework effectively identifies health conditions and recovery facilitators, leading to improved neuroplasticity and motor function through a patient-centered approach. The study also highlighted caregivers’ vital role and integration into the recovery process. These findings align with broader research emphasizing stroke recovery as an individualized and multifaceted journey [4].
- Rathi Ratha Krishnan et al. [5] analyzed functional outcomes in TBI patients of different ages in Singapore. Their retrospective study of 203 patients found that older adults, despite achieving significant recovery, had lower discharge FIM scores and higher dependency rates. The study highlights the critical role of early intervention and comprehensive care in mitigating age-related disparities in TBI outcomes. This is consistent with existing literature that underscores the need for tailored rehabilitation strategies to enhance neuroplasticity and recovery [6].
- In another study, Rathi Ratha Krishnan et al. [7] compared rehabilitation outcomes between stroke patients with Cerebral Infarction (CI) and Intracerebral Hemorrhage (ICH). Despite initial severity differences, both groups showed significant functional improvements, suggesting that ICH patients can achieve outcomes comparable to those with CI. The study also identified premorbid employment as a predictor of better discharge function, while the ICH subtype and lower cognitive FIM scores at admission predicted poorer outcomes. The findings suggested that, despite the acute complexity of ICH, patients can achieve comparable rehabilitation outcomes compared to CI patients, challenging the notion that hemorrhagic stroke survivors have poor functional prognoses.
- Poo Lee Ong et al. [8] examined functional outcomes in hemorrhagic stroke patients who were managed both surgically and non-surgically. Both groups showed significant improvements with no major differences in discharge FIM scores, though severe cases required extensive post-discharge care, underscoring the need for optimized rehabilitation strategies.
- Another study by Poo Lee Ong et al. [9] investigated unplanned acute care unit readmissions (ACURs) during TBI rehabilitation. They found that ACURs were associated with poorer functional outcomes and longer rehabilitation stays, highlighting the necessity for early complication management to enhance recovery. The study underscored the need for targeted interventions to minimize ACUR incidence and associated socio-economic burdens, emphasizing the critical role of tailored rehabilitation strategies in optimizing outcomes for TBI patients.
- Matthew Rong Jie Tay et al. [10] evaluated long-term outcomes in patients with primary brain tumors undergoing inpatient rehabilitation. Their study revealed significant functional improvements, particularly in those with lower-grade tumors, and emphasized the critical role of caregivers in supporting long-term recovery.
- Wei-Cheng Wang et al. [11] reviewed the combined effects of robot-assisted training and antispasticity therapy on stroke and spinal cord injury patients. The review found that combined therapy improved lower limb function but had inconclusive effects on upper limbs and spasticity, recommending standardized protocols and further high-quality studies. These findings support the growing body of evidence advocating for robotic rehabilitation in neurorehabilitation to enhance motor recovery [1,12].

These studies collectively highlight the importance of individualized, early, and comprehensive rehabilitation approaches in neurological recovery, demonstrating significant advancements and ongoing challenges in the field.

### 2.2. Rehabilitation Care During the Coronavirus Disease 2019 (COVID-19) Pandemic

The COVID-19 pandemic significantly disrupted rehabilitation care, introducing challenges that required major adjustments in patient management and healthcare practices. While the pandemic has subsided, its lessons underline the critical role of rehabilitation during health crises. This section highlights two studies examining the impact of COVID-19 on rehabilitation.

Karen Sui Geok Chua et al. [13] investigated the functional outcomes of TBI patients before and during the pandemic. Their findings revealed that post-COVID-19 TBI patients were older, had more comorbidities, and exhibited lower functional independence at discharge. The study identified a demographic shift toward frailer, older patients with more severe TBIs, resulting in slower and less pronounced recovery. There was also a notable increase in caregiver demand, with many patients transitioning to nursing homes or care facilities post-rehabilitation. These changes were attributed to pandemic-induced disruptions in healthcare practices, underscoring heightened healthcare and socio-economic challenges for TBI survivors. 

Zdeněk Guřan and colleagues [14] analyzed acute rehabilitation for COVID-19 patients hospitalized between March 2020 and December 2021 at the University Hospital of Ostrava. Among these patients, 16.6% required artificial pulmonary ventilation, 1.8% underwent ECMO, and 11.9% needed high-flow oxygen. Rehabilitation durations ranged from 1 to 102 days, with most patients (92.0%) receiving care for 1–15 days. The study highlighted the vital role of rehabilitation in enabling COVID-19 survivors to recover and return home while exposing challenges such as increased staffing demands, modified rehabilitation protocols, and strain on resources. Recommendations included strengthening rehabilitation infrastructures, improving staff training, and leveraging technology, like online rehabilitation, to enhance resilience in future health crises.

### 2.3. Cardiopulmonary Rehabilitation

Ming-Hsuan Huang et al. [15] conducted a retrospective cohort study to evaluate exercise capacity in patients with mitral valve prolapse (MVP) using cardiopulmonary exercise testing (CPET). The study included 45 MVP patients, and 76 matched healthy controls, assessing body composition, pulmonary function, and CPET metrics such as peak metabolic equivalent (MET), oxygen consumption (VO2), and peak rate pressure product (PRPP). Results showed no significant differences in peak MET, AT MET, or other CPET parameters between groups. However, MVP patients had significantly lower PRPP, suggesting potential coronary perfusion issues and subtle left ventricular dysfunction. Despite this, MVP patients displayed similar overall physical fitness to healthy controls.

I-Ching Huang et al. [16] assessed the oxygen uptake efficiency slope (OUES) as a predictor of one-year major adverse cardiovascular events (MACEs) in acute decompensated heart failure patients. Pre-discharge CPET was performed on 85 patients, revealing OUES as a stronger prognostic marker than traditional parameters such as peak VO2, VE/VCO2 slope, and 6MWT. Patients with OUES < 1.25 had a 5.421-fold increased risk of MACE. OUES, independent of peak respiratory exchange ratio, is particularly valuable for patients unable to achieve maximal effort during CPET, reinforcing its importance in guiding cardiac rehabilitation and risk stratification.

Anna Kubincová et al. [17] examined the effects of climatic rehabilitation on the quality of life in Chronic Obstructive Pulmonary Disease (COPD) patients using two Minimum Clinically Important Difference (MCID) thresholds in the St. George’s Respiratory Questionnaire (SGRQ). Involving 90 patients in a three-week program in High Tatras, Slovakia, the study incorporated respiratory physiotherapy, hydrotherapy, and climatotherapy. Significant improvements were observed in FEV1, 6MWT results, and anxiety and depression scores. Greater baseline limitations in pulmonary function and SGRQ scores predicted larger benefits. The study validated both 4-unit and 7-unit MCID thresholds for measuring outcomes and highlighted climatic rehabilitation as an effective strategy for COPD management.

### 2.4. Orthopedic Rehabilitation and Pain Management

Low-back pain (LBP) is a significant health issue that burdens both individuals and healthcare systems. Ida Kartini Othman et al. [18] used ultrasound and surface electromyography to investigate the relationship between piriformis thickness and the morphological and functional changes in gluteal maximus and medius muscles among 91 LBP patients with and without piriformis syndrome. These findings offer valuable insights into the muscular interactions in LBP, particularly in those with piriformis syndrome, and can assist clinicians in managing these conditions more effectively.

Yu-Chi Su et al. [19] conducted a meta-analysis to evaluate the effectiveness and safety of botulinum toxin A (BoNT) in managing complex regional pain syndrome (CRPS), which is characterized by pain, limited mobility, and other symptoms. Analyzing eight studies with 176 patients, including three randomized controlled trials, they found that BoNT significantly reduced pain at the first follow-up (3 weeks to 1 month post-treatment), though this effect diminished by 2–3 months. Adverse events were mild, temporary, and self-limited. While BoNT appears promising for CRPS pain relief, the variability in treatment protocols, small sample sizes, and high study heterogeneity suggest the need for larger trials to confirm its therapeutic efficacy and optimize its use. Their findings align with the growing interest in multimodal approaches for pain management that integrate pharmacological and physical rehabilitation strategies [20].

### 2.5. Innovative Technologies and Approaches

Advances in rehabilitation technologies, such as telerehabilitation, neuromodulation, and balance assessment tools, are transforming patient care by improving accessibility and personalization. These trends are reinforced by previous research on the role of digital health interventions in overcoming geographic barriers to rehabilitation access [21]. Three notable studies highlight these innovations:

Chernkhuan Stonsaovapak et al. [22] developed an eight-week telerehabilitation program for older adults in rural Thailand, combining tele-exercise sessions with wearable sensor assessments. Participants showed improved physical performance, walking ability, and high satisfaction, demonstrating the system’s potential to overcome geographic barriers. However, the absence of a control group limited the findings, and further randomized trials were recommended.

Ashleigh Peng Lin et al. [23] conducted a randomized, double-blind trial on high-definition transcranial alternating current stimulation (HD-tACS) for fibromyalgia. While HD-tACS reduced symptom severity and was well tolerated, its effects did not significantly outperform placebo samples. The study highlighted the need for optimizing stimulation parameters to enhance clinical efficacy.

Wen-Yen Liao et al. [24] used center of pressure (CoP) data to estimate Mini-BESTest scores for assessing balance and fall risk in elderly individuals. A decision tree algorithm outperformed linear regression, achieving 76.15% accuracy in classifying scores. These findings suggest CoP data combined with decision tree classification can provide a practical tool for estimating Mini-BESTest scores, aiding balance assessments in elderly patients reluctant to complete traditional questionnaires.

## 3. Emerging Trends and Future Directions

Rehabilitation care increasingly incorporates advanced technologies, personalized treatment protocols, and a holistic approach to patient needs. Trends such as telerehabilitation, wearable devices, and neuromodulation are addressing unique patient challenges and enhancing outcomes, particularly for conditions like TBI, stroke recovery, and fibromyalgia. Remote monitoring and advanced technology are bridging healthcare gaps, especially in underserved populations. Future advancements will focus on improving accessibility, streamlining treatment pathways, and refining interventions. Additionally, the emphasis on addressing psychological, social, and caregiver aspects highlights the need for a comprehensive approach to rehabilitation. As research progresses, greater integration of these strategies and the development of new methodologies will enhance long-term effectiveness and sustainability in care.

## 4. Future Research Needs

This Special Issue highlights the rapid evolution of rehabilitation medicine, showcasing innovations that integrate evidence-based practices, technology, and personalization to meet modern healthcare challenges. Future research should prioritize several areas:

**Long-Term Outcomes:** Conduct longitudinal studies to evaluate the sustainability of rehabilitation outcomes for chronic conditions like neurological impairments, TBI, and fibromyalgia.

**Validation of Emerging Technologies:** Perform rigorous trials to confirm the effectiveness of innovations such as telerehabilitation, HD-tACS, and robotic-assisted therapies across diverse settings and populations. 

**Multimodal Approaches:** Investigate the integration of pharmacological treatments with physical rehabilitation to create personalized, effective therapies.

**Health Disparities:** Address disparities by improving access to advanced rehabilitation for rural and underserved populations.

**Standardization:** Develop standardized metrics for evaluating rehabilitation outcomes to improve comparability across studies and clinical practices.

**Caregiver Involvement:** Study the role of caregivers and families in rehabilitation to design comprehensive support systems that enhance recovery and well-being.

These research priorities aim to advance rehabilitation practices, ensuring that they are effective, equitable, and sustainable for diverse patient populations.

## References

[1] Banyai A.D., Brișan C. (2024). Robotics in Physical Rehabilitation: Systematic Review. Healthcare.

[2] Cieza A., Causey K., Kamenov K., Hanson S.W., Chatterji S., Vos T. (2021). Global estimates of the need for rehabilitation based on the Global Burden of Disease study 2019: A systematic analysis for the Global Burden of Disease Study 2019. Lancet.

[3] Li S. (2023). Stroke Recovery Is a Journey: Prediction and Potentials of Motor Recovery after a Stroke from a Practical Perspective. Life.

[4] Langhorne P., Bernhardt J., Kwakkel G. (2011). Stroke rehabilitation. Lancet.

[5] Ratha Krishnan R., Ting S.W., Teo W.S., Lim C.J., Chua K.S. (2023). Rehabilitation of Older Asian Traumatic Brain Injury Inpatients: A Retrospective Study Comparing Functional Independence between Age Groups. Life.

[6] Matney C., Bowman K., Berwick D., National Academies of Sciences, Engineering, and Medicine, Health and Medicine Division, Board on Health Sciences Policy, Board on Health Care Services, Committee on Accelerating Progress in Traumatic Brain Injury Research and Care (2022). Traumatic Brain Injury: A Roadmap for Accelerating Progress.

[7] Ratha Krishnan R., Yeo E.Q., Lim C.J., Chua K.S. (2022). The Impact of Stroke Subtype on Recovery and Functional Outcome after Inpatient Rehabilitation: A Retrospective Analysis of Factors. Life.

[8] Ong P.L., Seah J.D., Chua K.S. (2023). Inpatient Rehabilitation Outcomes after Primary Severe Haemorrhagic Stroke: A Retrospective Study Comparing Surgical versus Non-Surgical Management. Life.

[9] Ong P.L., Rosiana A., Chua K.S. (2023). Characteristics and Functional Impact of Unplanned Acute Care Unit Readmissions during Inpatient Traumatic Brain Injury Rehabilitation: A Retrospective Cohort Study. Life.

[10] Tay M.R., Seah J.D., Chua K.S. (2022). Long-Term Outcomes of Patients with Primary Brain Tumors after Acute Rehabilitation: A Retrospective Analyses of Factors. Life.

[11] Wang W.-C., Yeh C.-Y., Huang J.-J., Chang S.-C., Pei Y.-C. (2023). Synergic Effect of Robot-Assisted Rehabilitation and Antispasticity Therapy: A Narrative Review. Life.

[12] Hong R., Li B., Bao Y., Liu L., Jin L. (2024). Therapeutic robots for post-stroke rehabilitation. Med. Rev..

[13] Chua K.S., Kwan H.X., Teo W.S., Cao R.X., Heng C.P., Ratha Krishnan R. (2023). Changing Epidemiology and Functional Outcomes of Inpatient Rehabilitation in Asian Traumatic Brain Injury Cases before and during the COVID-19 Pandemic: A Retrospective Cohort Study. Life.

[14] Guřan Z., Pastucha D., Sněhotová Z., Honzíková L., Maďar R., Tomášková H. (2023). The Role of Acute Rehabilitation during the COVID-19 Pandemic: A Retrospective Study in the Czech Republic. Life.

[15] Huang M.-H., Tuan S.-H., Tsai Y.-J., Huang W.-C., Huang T.-C., Chang S.-T., Lin K.-L. (2023). Comparison of the Results of Cardiopulmonary Exercise Testing between Healthy Peers and Pediatric Patients with Different Echocardiographic Severity of Mitral Valve Prolapse. Life.

[16] Huang I.C., Chen Y.-J., Chen C.-H., Huang W.-C., Lin K.-L. (2022). The Pre-Discharge Oxygen Uptake Efficiency Slope Predicts One-Year Cardiovascular Events in Acute Decompensated Heart Failure Patients. Life.

[17] Kubincová A., Takáč P., Demjanovič Kendrová L., Joppa P. (2023). Predictors of Quality-of-Life Improvement at Different Minimum Clinically Important Difference Values in Patients with Chronic Obstructive Pulmonary Disease after Climatic Rehabilitation Treatment. Life.

[18] Othman I.K., Raj N.B., Kuan C.S., Sidek S., Wong L.S., Djearamane S., Loganathan A., Selvaraj S. (2023). Association of Piriformis Thickness, Hip Muscle Strength, and Low Back Pain Patients with and without Piriformis Syndrome in Malaysia. Life.

[19] Su Y.-C., Hsieh P.-C., Guo Y.-H., Lin Y.-C. (2022). Meta-Analysis of Effectiveness and Safety of Botulinum Toxin in the Treatment of Complex Regional Pain Syndrome. Life.

[20] Almeida B.O., Barreto E.S.R., Antunes Júnior C.R., Alencar V.B., Souza A.K.d.N., Azi L.M.T.d.A., Lins-Kusterer L.E.F., Kraychete D.C. (2024). Evaluating the efficacy of botulinum toxin in treating complex regional pain syndrome: A systematic review. Toxicon.

[21] Borges do Nascimento I.J., Abdulazeem H., Vasanthan L.T., Martinez E.Z., Zucoloto M.L., Østengaard L., Azzopardi-Muscat N., Zapata T., Novillo-Ortiz D. (2023). Barriers and facilitators to utilizing digital health technologies by healthcare professionals. NPJ Digit. Med..

[22] Stonsaovapak C., Sangveraphunsiri V., Jitpugdee W., Piravej K. (2022). Telerehabilitation in Older Thai Community-Dwelling Adults. Life.

[23] Lin A.P., Chiu C.-C., Chen S.-C., Huang Y.-J., Lai C.-H., Kang J.-H. (2022). Using High-Definition Transcranial Alternating Current Stimulation to Treat Patients with Fibromyalgia: A Randomized Double-Blinded Controlled Study. Life.

[24] Liao W.-Y., Chu Y.-H., Liu F.-Y., Chang K.-M., Chou L.-W. (2022). Cutoff Point of Mini-Balance Evaluation Systems Test Scores for Elderly Estimated by Center of Pressure Measurements by Linear Regression and Decision Tree Classification. Life.

